# 681. Compliance with Surgical Care Improvement Project (SCIP) Antimicrobial Prophylaxis Metrics Before and After National Policy Discontinuation: A Nationwide, Retrospective Cohort Study

**DOI:** 10.1093/ofid/ofaf695.220

**Published:** 2026-01-11

**Authors:** Hillary J Mull, Samuel Golenbock, Jacquelyn Pendergast, Rory Ostrow, Dipandita Basnet, Marlena Shin, Ryann Engle, Kathryn L Colborn, Mary Hawn, Westyn Branch-Elliman

**Affiliations:** VA Boston Healthcare System, Boston, Massachusetts; VA Boston Healthcare System, Boston, Massachusetts; VA Boston Healthcare System, Boston, Massachusetts; VA Boston Healthcare System, Boston, Massachusetts; VA Boston Healthcare System, Boston, Massachusetts; VA Boston Healthcare System, Boston, Massachusetts; VA Boston CHOIR, Boston, Massachusetts; University of Colorado, Aurora, Colorado; Stanford University, Palo Alto, California; Greater Los Angeles VA Healthcare System, UCLA David Geffen School of Medicine, Los Angeles, CA

## Abstract

**Background:**

SCIP measures included appropriate discontinuation of antimicrobial prophylaxis (AbxPPx) within 48 hours for cardiac surgeries. Metrics were manually collected and publicly reported; over time, the program was associated with compliance rates > 95% in Veterans Health Administration (VHA) hospitals. Manual review is expensive and SCIP was discontinued in 2015. The objective of this study was to measure compliance with SCIP INF-3, timely discontinuation of AbxPPx, in VHA cardiac surgery to assess whether compliance was sustained after the active reporting period.

**Figure 1.**
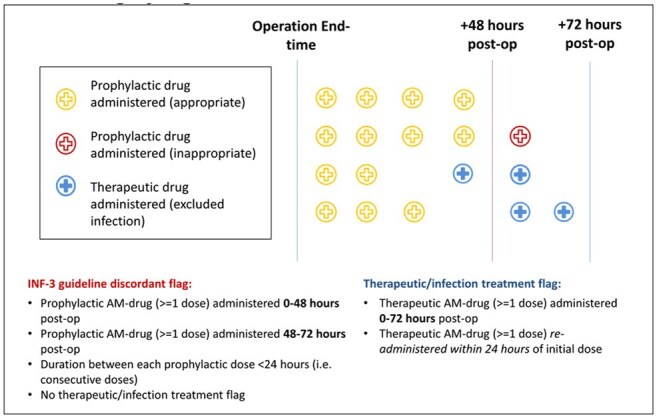
Timing of SCIP INF-3 compliance, discordance, and exclusion criteria for cardiac surgery algorithm

**Figure 2.**
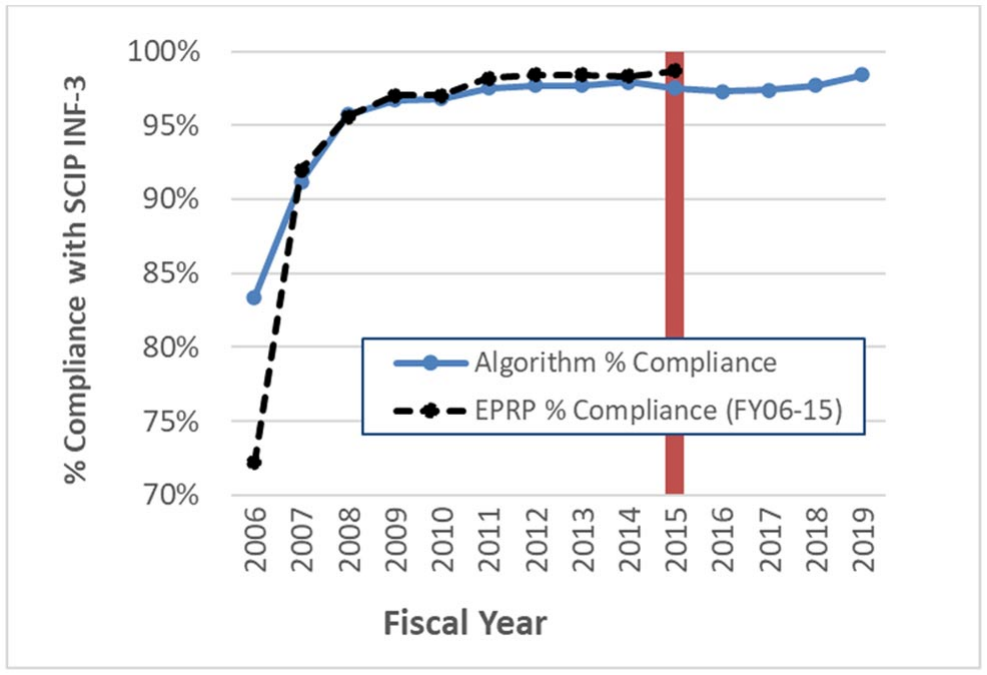
Incidence of SCIP INF-3 compliance (appropriate discontinuation of post-op antibiotics) in EPRP-reviewed data (n=33,036, 2006-2015) vs. algorithm among eligible CDW cardiac surgeries (n=56,244, 2006-2019)

**Methods:**

This retrospective cohort study of cardiac surgeries from 2006 -2019, included data from the Corporate Data Warehouse (CDW) and manually reviewed AbxPPx compliance data from VHA External Peer Review Program (EPRP) from 2006-15. CDW cardiac surgeries were merged with EPRP data to develop a robust electronic algorithm to measure SCIP INF-3 compliance (Fig 1). The algorithm was applied to cases from 2011-2019 to assess whether SCIP INF-3 compliance was ≥ 95% after public reporting was discontinued. Sustainability was assessed by performing an interrupted time-series analysis with 2015 SCIP discontinuation as the interruption. The Poisson regression model with time offset estimated change in slope immediately at discontinuation and annually over time in the pre- and post-SCIP periods, controlling for facility random effects.

**Figure 3.**
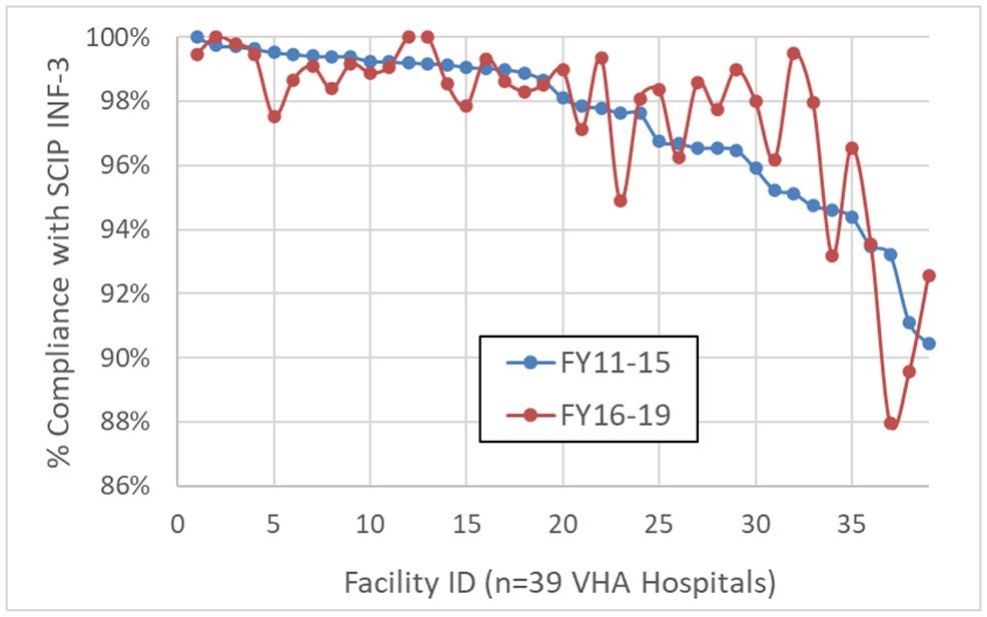
Facility-level SCIP INF-3 compliance rates during SCIP active reporting and after discontinuation

**Results:**

SCIP INF-3 compliance as assessed by EPRP (98.4%) and the electronic algorithm (97.7%) are presented in Fig 2. Among eligible cardiac surgeries performed at 39 hospitals, AbxPPx compliance rates did not change from 2011-2019; however, the time series model identified a slight and significant dip in compliance the year that SCIP was discontinued. Individual facilities were relatively consistent in compliance rates during SCIP active reporting and after discontinuation, suggesting stable practices over time. In some cases, facility-level SCIP INF-3 compliance improved over time (Fig 3).

**Conclusion:**

SCIP sustainably improved AbxPPx use for major cardiac surgeries. Future work should evaluate ongoing compliance other specialties and whether AbxPPx guidelines issued during the SCIP reporting period were broadly implemented.

**Disclosures:**

Westyn Branch-Elliman, MD, MMSc, DLA Piper, LLC/Medtronic: Advisor/Consultant|DLA Piper, LLC/Medtronic: Expert Testimony|Shiongi: Advisor/Consultant

